# Real Time Voltage and Current Phase Shift Analyzer for Power Saving Applications

**DOI:** 10.3390/s120811391

**Published:** 2012-08-21

**Authors:** Ondrej Krejcar, Robert Frischer

**Affiliations:** Department of Information Technologies, Faculty of Informatics and Management, University of Hradec Kralove, Rokitanskeho 62, Hradec Kralove 50003, Czech Republic; E-Mail: Robert.Frischer@gmail.com

**Keywords:** remote sensors, analyzer, real-time measurement, phase shift

## Abstract

Nowadays, high importance is given to low energy devices (such as refrigerators, deep-freezers, washing machines, pumps, *etc.*) that are able to produce reactive power in power lines which can be optimized (reduced). Reactive power is the main component which overloads power lines and brings excessive thermal stress to conductors. If the reactive power is optimized, it can significantly lower the electricity consumption (from 10 to 30%—varies between countries). This paper will examine and discuss the development of a measuring device for analyzing reactive power. However, the main problem is the precise real time measurement of the input and output voltage and current. Such quality measurement is needed to allow adequate action intervention (feedback which reduces or fully compensates reactive power). Several other issues, such as the accuracy and measurement speed, must be examined while designing this device. The price and the size of the final product need to remain low as they are the two important parameters of this solution.

## 1. Introduction

Energy savings are a common issue of everyday life. As energy prices increase, devices that can reduce the energy consumption become commonplace. The devices currently available on the market are either relatively simple (in terms of functionality) or very complex. However, the efficiency of energy saving solution is directly proportional to the accuracy of the acquired quantities (voltage and current). The knowledge of these quantities is essential for successive processing. As a solution an analyzer device can be developed to gather the passing current and terminal voltage and to perform basic data processing.

Voltage measurement is relatively simple and depends on the complexity of the successive saving device which needs to be precise. Then the true Root Mean Square (RMS) voltage value measurement is relatively problematic. If the voltage has no sinusoidal profile and contains irregular spikes, cheap Integrated Circuits (ICs) with low sampling frequencies may produce inaccurate values [1].

Current measuring has similar problems. It remains correct while sinusoidal running is observed. Additionally more complex saving devices need precise voltage and current running, not only their RMS value. The aim of this paper is to present a proprietary analyzer we have developed, which is able to satisfy all needs in the power saving area.

## 2. Problem of Quantities and Their Measuring

The basic parameter that we need to measure is the amount of reactive power in the power lines. It is the main component which overloads power lines and produces excessive thermal stress on conductors. Reactive power (mostly of an inductive character) is also undesirable for power providers. The decrease of reactive power leads to lower expenses. There are two existing options to compensate it:

1. It can be measured as the total amount of reactive power when a suitable capacitor can be consequently added to compensate this reactive power.
2. It can be also measured by the phase shift between voltage and current which can be solved by gradually connected capacitors (in parallel).

Knowledge of the exact voltage and current in real time is also desirable for technicians who must produce the final evaluation of target buildings or companies where an estimate of the approximate value of savings is needed. Other features are derived from these measurements.

## 3. Related Work

In [2] the authors are solving the reactive power presence issue. The great importance of reactive power measurement is mentioned in the introduction. Various types of energy meters and the influence of harmonic distortion on measurement accuracy are also mentioned in the article. The main difference between the discussed devices and required solution is that an exact sinusoidal performance is not needed. This is the essential condition which has to be observed in standard energy meters. Non-sinusoidal voltage and current running are common when driving load through thyristors or triacs. This article is a positive start when studying information about energy metering.

In another paper [3], the authors described a device which is intended to drive compensation equipment. The main idea is very close to the solution that we require, but the design is obsolete. The described device is only a “driver” with an analyzer core. Zero cross detection was used as a solution, but without small differences which make it less robust and not immune to glimmers or disturbances. It is obvious that creators are skillful while working with hardware, but do not have sufficient experience. Nevertheless, their solution is the closest to the required one. We need a device which can also be used to drive compensation devices, not only on one level (compensation or not) but on more levels.

Reference [4] is a continuation of [3], but from other authors. Their solution presents a professional design. They measure all quantities by microprocessor and do all necessary calculations in an MCU. Since the sensing circuits are more complex they also need complex software in the processing MCU. The authors use voltage transformers to measure high voltage on the primary side, but it is not suitable because of distortion which they add to the signal and the phase shift (minimal, but measurable) between real voltage running and measured voltage running. This solution is only for sensing reactive, real and apparent power and displaying it on connected LCD.

Reference [5] is also closely related to a driving Power Factor Correction (PFC) device. They are sensing input voltage and current and evaluate the state of energies in a circuit. If there is enough reactive power, they will connect an inductor (through the triac) and then will be able to improve PFC (one level driving).

These references were carefully studied and the conclusions were derived. No one can store measured data for post processing. All of them are using measured or calculated data for immediate action control and then discard them. A solution is needed which will be able to store these data and use them for subsequent processing or for improving the power line condition or finding mistakes or bad devices in this circuit. Table 1 summarizes the main differences of all compared solutions while it also defines the needed solution.

There are also many other devices which cannot display immediate running values of important quantities, but they can store these values for a long time for subsequent post processing. One of these devices is for example the Janitza Electronics Power Quality Management device [6]. This device is primarily intended for monitoring reactive power in the power lines and then driving another device which will provide operational intervention.

A similar product is the HIOKI Power Quality Analyzer [7]. It is a complex device which can analyze any of the important quantities and it can store data in its internal memory for later display. This is a professional device with a corresponding “professional” price of about 5,000 USD. For an electrician, it is unthinkable to buy more of these devices to analyze several different buildings or areas. This device is intended for other purposes.

There are several other models on the market, but their price is too expensive for various types of utilization (as for mentioned cases at home or small companies' usage). Generally they are mainly mobile, two channel oscilloscopes, which can display the phase shift between voltage and current and are capable of estimating global power line quality. If we additionally want to save passing data, we need to spend more than $1,500 per device.

The best solution would be to use a cheap, simple to manage data recording analyzer, which is unfortunately currently not on the market. This led us to develop such a unit—an analyzer device which must determine:

- phase shift of voltage and current,
- their RMS values,
- maximum and minimum values,
- average value (to detect asymmetry in the power line),
- reactive power amount,
- active power amount,
- apparent power amount,
- time, and others.

In addition to the previous solution, the developed solution needs to provide the possibility of recording these values onto a removable SD card.

## 4. Analyzer Design

**Figure 1.**
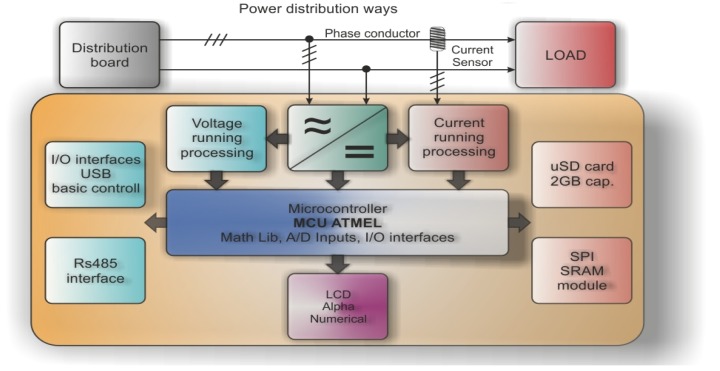
Analyzer block diagram.

All of the mentioned issues were taken into account during the development phase of our new analyzer design (Figure 1). The analyzer is mounted inside the home or company distribution board box.

Voltage probes are screwed directly to the voltage terminals while current probes are deployed onto the phase conductor. Current probes are standard pincer type with a current ratio of 3,000:1. Power is delivered through the standard, Printed Circuit Board (PCB) mounted transformer with single output voltage. Nevertheless there is some novelty, which is the shifted ground signal. This is caused by the operational amplifiers that have to be driven by a slightly negative voltage against the signal ground. These adjustments are made to obtain the maximum resolution of scanned quantities which has an amplitude from almost Vdd to Vss. Without the negative operational amplifier driving voltage, the lowest signal running will remain undetectable (because of operational amplifier output swing). Even the best RailToRail operational amplifier cannot drive its output from Vss to Vdd. In order to satisfy the above mentioned needs, the voltage and current have to be sampled and processed in real time. After some tests, 400 point period resolution (X axis) and 10 bit A/D resolution (Y axis) [8] was chosen.

In case of a three phase system analysis, we need to store 400 × 2 × 3 samples (2-current and voltage; 3-3 phases). This results in 2,400 samples × 10 bit = 4800 bytes of RAM memory. This is a relatively high number which led us to use external SRAM IC communicating over the SPI interface [9]. To simplify and speed up the process of reactive power evaluation, a hardware circuit (which can detect passage through the zero for voltage and current running) was added. By doing this, it is possible to precisely define the shift between these two quantities. Ideally the shift is equal to zero. This happens only if resistive load is present. All current is then utilized to create real power (unfortunately a sporadic occasion). In most cases, some inductive load always exists (capacitive load is rare).

**Figure 2.**
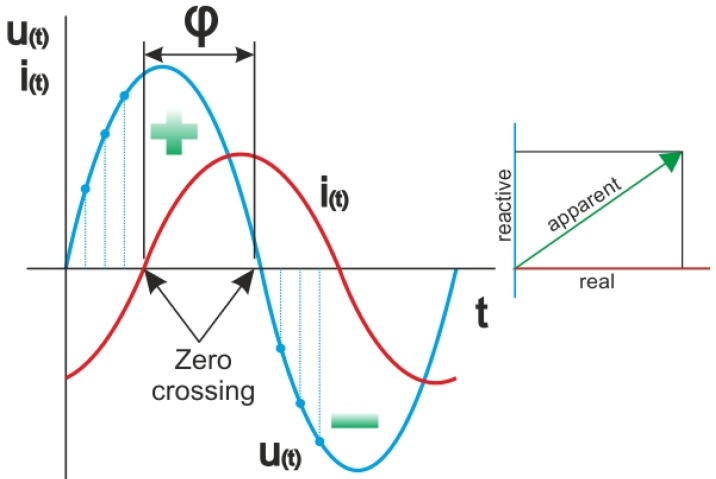
Determining phase shift between current and voltage.

If a phase shift φ has been determined (Figure 2), we can derive other parameters like reactive power and apparent power using simple formulas [10]. It is a vector sum of reactive and real power [11]. RMS values of voltage, current and powers cannot be calculated by standard formulas due to the discrete area. These formulas have to be modified to passing from continuous to discrete area. Standard RMS value can be calculated by Equation (1):

(1) $$U_{\text{RMS}}=\sqrt{\frac{1}{T}\int_{t0}^{t0+T}u^{2}(t)dt}$$

where:
*U_RMS_*:Root Mean Square Voltage value ↕ continuous*T*:ac voltage period*t*0:Starting time*u*:Voltage value in defined time*t*:Time ↕ <0;T>

However this formula does not mention a disposition of continuous value of voltage. Passing to discrete area, an integral will change to the sum and the resulting formula's shape will be as follows:

(2) $$U_{\text{RMS DISCRETE}}=\sqrt{\frac{1}{n}\sum_{i=1}^{n}u_{1}^{2}}$$

where:
*U_RMS DISCRETE_*:Root Mean Square Voltage value ↕ discrete*i*:Current sample number*n*:Total amount of samples in one period*u_i_*:Voltage value in defined sample

Apparent power can be then expressed as:

(3) $$S_{\text{DISCRETE}}=u_{\text{RMS}}\cdot i_{\text{RMS}}$$

where:
*S_DISCRETE_*:Apparent power*u_RMS_*:Actual voltage value*i_RMS_*:Actual current valueand active power as follows:

(4) $$P_{\text{DISCRETE}}=\frac{1}{n}\sum_{i=1}^{n}u_{i}\cdot i_{i}$$

where:
*P_DISCRETE_*:Real power*i*:Current sample number*n*:Total amount of samples in one period*u_i_*:Voltage value in defined sample*i_i_*:Current value in defined sample

Finally reactive power value is calculated by simple statement:

(5) Q=S−P

where:
*Q*:Reactive power*S*:Apparent power*P*:Real Power

Variable n is a number of samples (in our case 400). These formulas are easy to implement into the MCU used while results are sufficiently exact. Moreover, the searching for maximum values of voltage and current is important, because these values may indicate some irregularities in the power line which have to be carefully taken into account. It is easy to find them, if we have a row of measured samples Equation (6).

(6) $${\left(\text{IF}(u_{i}>\max)\to \max=u_{i}\right)}_{i=1}^{n}$$

where:
*u_i_*:Voltage value in defined sample*max*:Variable „max‟*n*:Total amount of samples in one period

Presence of peaks is also very important due to the fact that in the case of non-sinusoidal running of voltage and current the standard analyzers cannot be used for their insufficiency. Finally, the existence of current spikes which are very dangerous to connected active switching devices is problematic. These events can be analyzed by a connected computer device (e.g., PC) where it is possible to display one complete recorded period (20 ms). The attached LCD display is mainly suitable for basic information as it only shows the main and most important values. The PC is remotely connected via RS485 interface where the display of one period of current and voltage takes only 100 ms (from time of measurement).

**Figure 3.**
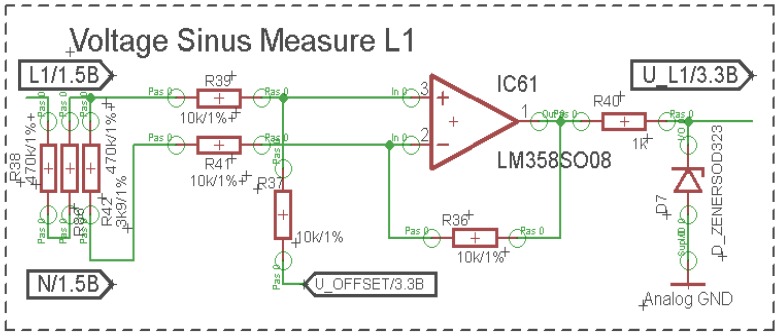
Input voltage level measuring.

The main goal of the analyzer application was to display an amount of savings for a specified building or company. This was done by analyzing reactive power, total active power, shape of current running and terminal voltages. Voltage measurement was the easiest one. The resistor divider was applied on the input of differential operational amplifier (Figure 3).

Input voltage was divided by the factor of 241 and shifted up of 1.8 V to sustain the ability to measure the positive and negative part of the sinus wave. Total voltage gain was equal to 1. Input resistance stayed high (1 MΩ) to prevent unwanted crosstalk.

On the contrary, current measurement is a greater problem than the previous case. Although there are many ways to measure the passing current, the easiest way is to insert a shunt resistor in the power line. Problems can arise when we need the target amplitude to be equal to 1 V. Then the power dissipation is enormous. Current is converted directly to heat energy where its amount can be expressed by formula (7):

(7) $$P_{\text{dissipation}}=i_{\text{RMS}}^{2}\cdot R_{\text{shunt}}=\frac{u_{\text{RMS}}^{2}}{R_{\text{shunt}}}$$

where:
*P_dissipation_*:Dissipating power*i_RMS_*:Actual current value*R_shunt_*:Shunt resistance*u_RMS_*:Actual voltage value

Based on real cases there is a need to measure a current of about 25 A, so a shunt resistor with value of about 40 mΩ is required. Total power dissipation is in this case is equal to 25 W of heat energy. This is too much heat energy to dissipate to the ambient environment without a large heat sink. For this reason this option could not be implemented.

**Figure 4.**
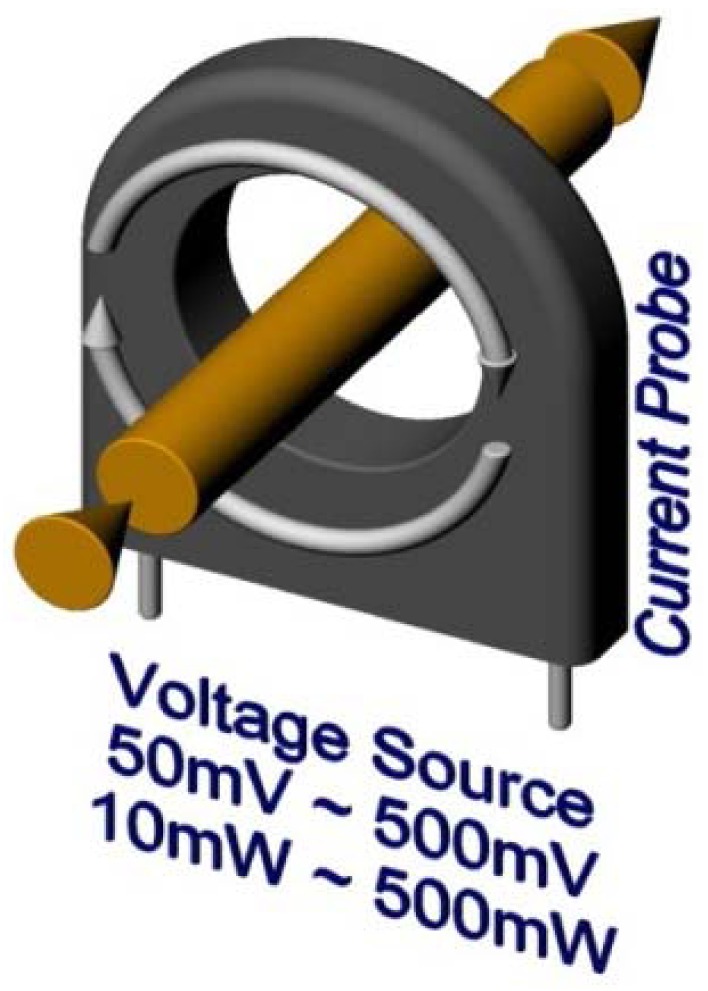
Current probe shape and basic features.

Another option is to measure magnetic field based on the Hall Effect, although the used sensors are relatively expensive [12]. Finally, the most efficient way to measure the passing current is to use a current transformer. It is a widely used cheap component with a very simple structure. The passing of AC current forms an alternating magnetic field which induces voltage on the secondary winding. This is a contactless measurement form, because the wire acts when the primary winding and magnetic lines are closing through the sensors winding (Figure 4).

**Figure 5.**
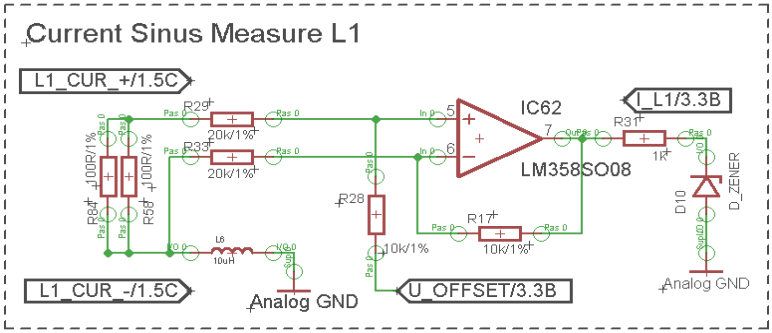
Current measurement and its adapter circuit.

On the probes terminal the same voltage pattern as the passing current is presented (only proportionally smaller). Of course, there is a small shift between real current and the current recorded by the probes, but if proper components are used, this error is smaller than 1%. This wave is also differentially scanned and shifted up to sustain the ability to measure both half waves (Figure 5).

**Figure 6.**
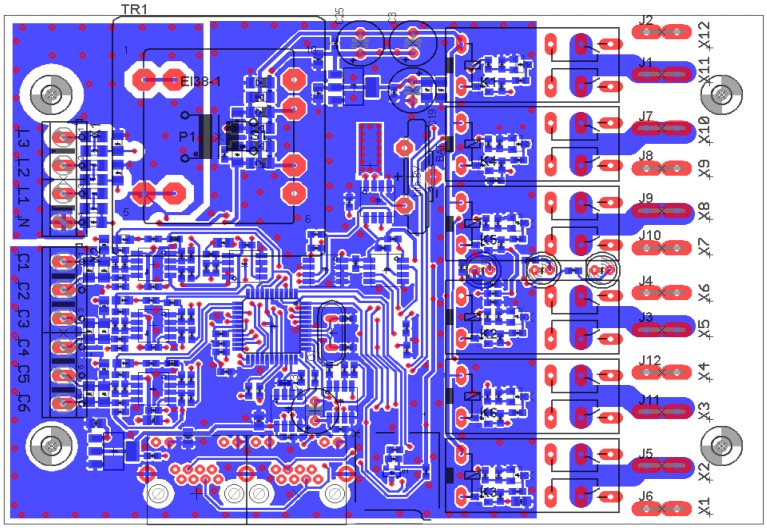
Analyzer PCB layout. Notice the spill GND signal.

To provide reasonable accuracy, input resistors must stay in ±0.5% accuracy tolerance. The operation amplifier inputs are again in a differential connection because of floating ground. Special care must be also targeted at PCB design where unwanted crosstalk is ubiquitous. Any conductor acts as an antenna, in this case because of the use of a relatively high AC voltage. It is mandatory to spill over the GND (ground connection) and to ensure that any unwanted noise is drained to ground. The PCB layout is presented in (Figure 6).

There the spill over the ground signal can be seen. Any parts which are concerned with a high AC voltage are also tightened together as close as possible (terminal N-L1-L2-L3 and resistors behind them). This is the only way to preserve a useful signal over the ubiquitous AC noise. The Real Time Clock (RTC) crystal even has a separate ground that is very susceptible to any disturbing impact where the shielding is the primary goal to ensure its flat running (small rectangle with many holes along the edge).

## 5. Visualization the SW Design

**Figure 7.**
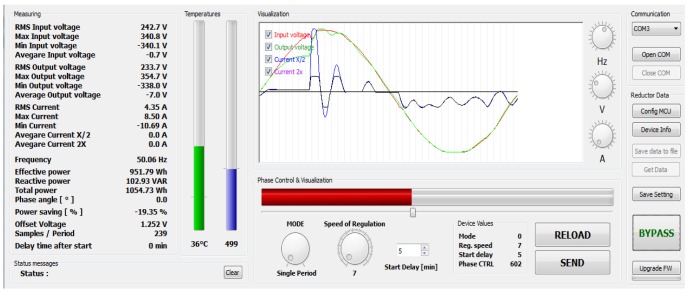
User software data visualization. Input and output sinusoidal voltage and passing current are displayed in the inner window.

Gathered data can be displayed when connected to a personal computer via a RS485 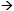 USB converter. There are many variables which are being transmitted. For visualization and setup purposes special software was designed which is able to recognize the input data string (Figure 7). Basically it is a software oscilloscope which is able to compare input and output voltage (red and green curves) and passing current running. This software is designed for online—onsite analysis. It provides valuable information about the processes inside the power line.

Gathered data are stored onto a microSD card. The second software was developed for visualization of these data and post processing analysis. Detailed information is available in Section 6.

Considering the example shown in Figure 7 the current has two amplification modes:

1. Small currents may be multiplied by two and then display better current running (dark pink curve).
2. High currents are on the contrary are divided by two which is why it they be displayed without distortion caused by saturation of the operation amplifier (blue curve). Multiplying and dividing are provided at the hardware level by setting of appropriate resistors.

This example (Figure 7) is from measuring street lightning. The current has odd running, because of the nature of High Intensity Discharge (HID) lamp drivers. This software can be used by service engineers where many other options (speed of regulation, regulating mode, device's temperature, offset voltage, RMS, maximum and minimum voltage levels, *etc.*) can be used.

## 6. Testing of Developed Analyzer Device in Real Environments

The test phase is a very important part of any device's development. Testing of devices in a real environment is the most suitable way. During the test phase we obtained strong feedback which led us to improve the first prototype. With small improvements, the device is now fully operational and ready to be set up in a real environment.

**Figure 8.**
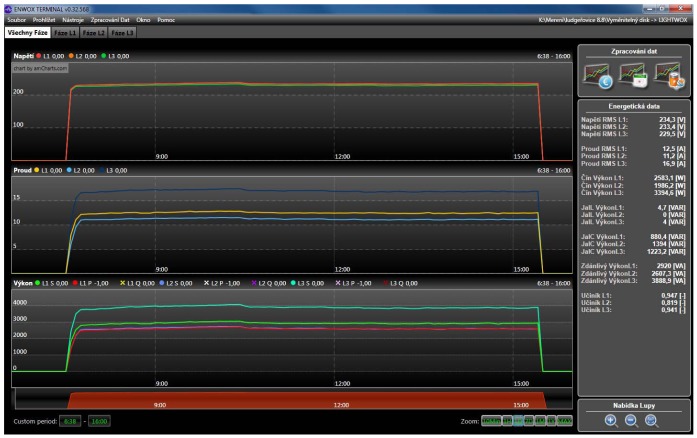
Main screen of the analyzing software. All necessary information is displayed. Minor information can be accessed through the additional icons and functions.

Analyzer devices are often used to analyze street light power lines. These circuits are relatively steady, therefore the voltage and current running are easy to analyze. The main problem in this application area is that too much reactive power is present [13]. It is a major problem which has to be resolved, because of the excess current strain of wires. Too much reactive power leads to overheating of the wires which causes essential loses and decreases the overall efficiency of the lightning process [14]. In Figure 8 a post processing software environment can be seen. Recorded data are processed and displayed. Various calculations are added for simple final evaluation. On the right side average values of main quantities are displayed, including all types of power and overall Power Factor Correction (PFC). In this figure one street lights' period is currently shown. On the top the voltages are applied to all phases where in the middle part the currents are shown. Finally, a power (optionally Reactive, Apparent, Real) are displayed at the bottom.

**Figure 9.**
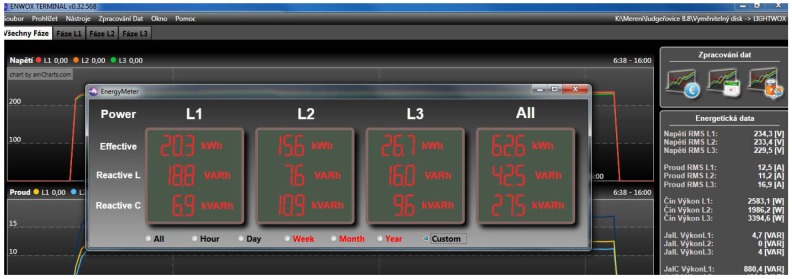
Power analysis is useful in case burned power in defined time period is needed, or if an extrapolation for a whole month or year is required.

As the next step in the analysis process a “Power analysis” can be run which provides information about burned power (Figure 9).

**Figure 10.**
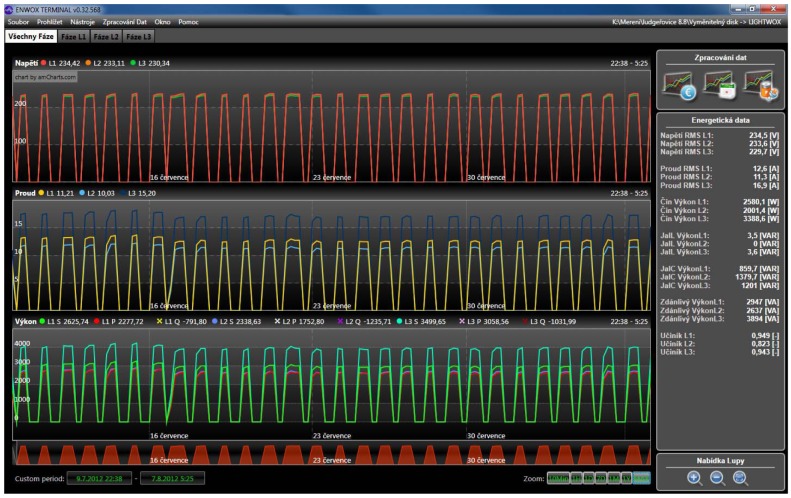
All recorded data are displayed on the main screen. There is also a switching ON and OFF of street lights which helps to provide an overview of the analyzed power line.

Data can be zoomed in and out; they can be printed and compared and so on. All data files are presented in Figure 10. In this case peaks of street lights are shown which are switched ON and OFF during its working period. The total power is slowly, day by day, decreasing, which can mean that used HIDs are aging and gradually fading.

## 7. Discussion

The created device was designed for analyzing power line circuits. The developed solution consists of three main components. Hardware is designed with the latest knowledge in the microelectronics area so the device is not prone to disturbances or spikes in the power line. The whole design uses Surface Mount Technology (SMT) which leads to a minimum wire length as well as minimum added noise and crosstalk.

The user software for immediate analysis can display online voltage and current running. This is very useful when searching for some anomalies like high current or voltage spikes, nonlinearities, power factor correction states or actual values of all main quantities. This software is for “in place” analysis. Startup currents and changes in reactive power after the transient can be observed there. This feature uses only of one device from the research described in [4] and one professional commercial device described more in Section 3 “Related Work”.

Power analysis software is for post processing. Data are continually stored on a microSD card so they can be further processed and evaluated. This software can calculate burned power and amount of reactive power. It can also display current and voltage levels over a selected period. It is possible to make extrapolations and interpolations of the main data which can help when reaching global conclusions and so on. The mentioned option of data recording is only provided on professional commercial devices as described in Section 3 “Related Work”.

The ability to drive subsequent power circuits is another very useful feature [15] and [16]. The devices described in [5] and [3] have this feature, but in very limited form. These solutions provide only one or three compensation levels which is very limiting. Using our solution from 4 to 128 compensation levels can be achieved (four in three phase operation, 128 levels in one phase operation respectively). This is a very helpful feature, because the online analyzer is always one logical step forward in operation.

## 8. Reduction of Reactive Power by a Reductor Device

The aim of this paper was the description of a developed analyzer device suitable for real time precise measurement of reactive and real power in power lines. However, the detection of reactive power is only the first step in the solution to reduce electric consumption. Another device named “Reductor” is needed to compensate this unwanted component of electric power. As Reductor, a robust and heavy transformer-based energy saver (reducer) or modern lightweight cheaper device can be used. This device can use active semiconductor switching topology to reduce RMS voltage. Smooth transitions of output voltage make it ideal to drive any kind of lights (including street lights). Any change is not noticeable which is what customers want. Also the price can be much lower than in current markets. Development of such a device is the next milestone of our current research.

## 9. Conclusions

The presented paper deals with a special analyzer design where several important issues were solved. This analyzer is intended to be used in low cost applications where expensive single-purpose analyzers are not an option.

As it was mentioned above, the developed solution is intended for every day measuring where it can satisfy the needs of on-site analysis and also in post processing at a working office. This solution is much smaller than other professional products. This was also one of the main requests for the device's development, because the device should be embedded in a distribution box where it is used to record passing currents, voltages and other useful quantities. The goal was not to develop the most accurate device. Accuracy is a very expensive feature and for the final evaluation of power lines it is not necessary. The developed device is able to measure voltage and current with <1% accuracy which is very good for the defined purposes (Section 2). Another development tends to use 32-bit processors, powerful ARM cores as well as external A/D converters. When used it can be compared favorably with any other commercial device (concerning accuracy). Other competitors are either accurate, good for onsite analysis, but without the ability to gather data or they are less accurate but intended for long time data gathering. The combination of both mentioned factors is not available in the market.

The developed software application provides all necessary and useful information on which basis the most suitable reducer can be applied to effectively drive power stage and to compensate reactive power to the minimum level on the basis of online measurement of operational quantities.

## Figures and Tables

**Table 1.** Related work functions summary.

**Reference**	[2]	[3]	[4]	[5]	**Needed Solution**
Real/Appar./React. Power	Yes/Yes/Yes	Yes/Yes/Yes	Yes/Yes/Yes	Yes/No/Yes	Yes/Yes/Yes
CPU processing	No	Yes	Yes	Yes	Yes
Ability to drive consequent devices	No	No	No	Yes	Yes
Data Record	No	No	No	No	Yes
Post processing	No	No	No	No	Yes
Technological level	Theory	Medium	Higher	Medium	High
Sinusoidal voltage need	Yes	Yes	Yes	Yes	No
Compensation levels	-	3	-	1	4-128
Three-phase operation	-	No	Yes	No	Yes
